# The serial changes of Neutrophile-Lymphocyte Ratio and correlation to weight loss after Laparoscopic Sleeve Gastrectomy

**DOI:** 10.3389/fsurg.2022.939857

**Published:** 2022-09-06

**Authors:** Po-Jui Chi, Kun-Ta Wu, Po-Jen Chen, Chung-Yen Chen, Yu-Chieh Su, Chung-Yi Yang, Jian-Han Chen

**Affiliations:** ^1^ Division of Nephrology, Department of Medicine, E-DA Hospital, Taiwan; ^2^ School of Medicine, College of Medicine, I-Shou University, Kaohsiung, Taiwan; ^3^ Division of General Surgery, Department of Surgery, E-Da Hospital, Taiwan; ^4^ Division of General Surgery, E-Da Cancer Hospital, Kaohsiung, Taiwan; ^5^ Department of Medical Research, E-Da Hospital, Taiwan; ^6^ Bariatric and Metabolism International Surgery Center, E-Da Hospital, Kaohsiung, Taiwan; ^7^ Division of Hematology-Oncology, E-Da Hospital, Kaohsiung, Taiwan; ^8^ Department of Medical Imaging, E-Da Hospital, Kaohsiung, Taiwan

**Keywords:** maximize effect, neutrophil-lymphocyte ratio (NLR), chronic inflammation, laparoscopic sleeve gastrectomy, immunology, prediction, adequate weight loss, morbid obesity

## Abstract

**Purpose:**

This study aims to identify the pre- and postoperative changes in the neutrophil-lymphocyte ratio (NLR) and its correlations to clinical characteristics in obese patients who underwent laparoscopic sleeve gastrectomy (LSG).

**Method:**

Retrospectively, we included patients who has undergone LSG in our institution between January 2019 and April 2021. A total of 100 patients whose body mass index over 32.5 and received primary laparoscopic sleeve gastrectomy without infectious condition were included.

**Results:**

There was a significant decline in NLR (T0 vs. POM3 2.21 vs. 1.78, *p* = 0.005), neutrophil (T0 vs. POM3 5369 vs. 4050, *p* < 0.001) and lymphocyte count (T0 vs. POM3 2440: 2100, *p* < 0.001, respectively) at postoperative 3 months (POM3) compared to preoperative (T0) levels, but similar between POM3 and POM6. The declined counts (Neutrophile vs. Lymphocyte 1445.5/µl vs. 323.5/µl, *p* < 0.001) and percentage (Neutrophile vs. Lymphocyte 25.11% vs. 13.07%, *p* < 0.001) of neutrophile are higher than lymphocyte from T0 to POM3, but similar in POM3 and POM6. Preoperative NLR has a significant correlation with the preoperative body weight, preoperative insulin level, and excessive body weight loss (EBWL) at POM3. Preoperative NLR <2.36 had a sensitivity of 67.6% and a specificity of 62.5% in predicting successful weight loss (EBWL > 37.7%) at POM3 (AUC = 0.635, *p* = 0.032).

**Conclusion:**

There was a significant decline in NLR, neutrophil, and lymphocyte count from T0 to POM3, but similar between POM3 and POM6. The declined counts and percentage of neutrophile are higher than lymphocyte. Preoperative NLR shows the potential to be used as a prognostic biomarker for predicting successful weight loss at POM3 after LSG. Further studies could be designed to evaluate the value of prediction in successful outcome after LSG and figure out the relationship between the changes of neutrophil function and oncogenesis.

## Introduction

Low-grade subclinical inflammation plays a pivotal role in the development of obesity. This is initiated by the activation of innate and adaptive immune cells resulting in an increased secretion of proinflammatory cytokines such as IL-1β, IL-12, IL-18, and IFN-*γ*. The development of the tumor is triggered by a variety of immune cells, including macrophages, neutrophils, dendritic cells, natural killer (NK) cells, and T and B lymphocytes(1) which is also assoicated with low-grade subclinical inflammation. The neutrophil-lymphocyte ratio (NLR), which is the ratio of neutrophils to lymphocytes in the peripheral blood, is a well-known indicator of subclinical inflammation. An elevated NLR has been associated with poorer prognoses in ischemic coronary disease, heart failure, peripheral vascular disease, and patients with obesity (2, 3). Higher NLR is significantly associated with an adverse overall survival (OS) in many solid tumors (4). NLR and IL-6 serve as a reliable biomarker combination for predicting tumors especially solid tumors (5). Moreover, a higher NLR was associated with an increased risk of breast cancer risk in the overall population (6). However, the actual mechanism regarding the function of NLR in systemic inflammation is still unknown (7).

Obesity is caused due to metabolic syndrome, initiates a chronic inflammatory state within adipose tissue depots, and is partly responsible for adipocyte dysfunction. Adipose dysfunction in obesity includes secretions of abnormal levels of cytokines which are linked to insulin resistance, impairments in triglyceride storage, and an increase in lipolysis. Subcutaneous adipose tissue (SAT) increases macrophage recruitment and contributes to the increased cellular inflammation and subsequent production of biomarkers that are associated with both insulin resistance and low-grade inflammation (8, 9). Obesity affects both T-cell-mediated and B-cell-mediated immune responses by altering cytokine synthesis, reducing antigen-specific responses, and impairing the functions of natural killer cells, dendritic cells, and macrophages (10–12), leading to an increased risk of infectious diseases. Obesity is proven to be a risk factor for various infections (13, 14), including respiratory tract infections (RTIs) such as pneumonia (15) and influenza (16, 17). Even after controlling for comorbidities, social behaviors, and sociodemographic factors, morbid obesity is associated with an increased risk of sepsis (18) and surgical site infections in both clean and contaminated wounds (19).

Bariatric surgery (BS) is currently one of the most effective therapeutic modalities for morbid obesity. BS significantly enhances weight loss, improves glucose metabolism, and decreases levels of systemic inflammation biomarkers. Baseline NLR was reported to be strongly correlated with postoperative 30-day outcome (20), as well as diabetes remission at postoperative 1-year and 5-year (21). A higher NLR was observed in patients with obesity with higher insulin resistance when compared to healthy individuals (22). However, only a few studies have demonstrated the association between weight loss and NLR. Moreover, several adaptive immunity changes have been reported, including a reduction in CD4+/ CD8+ T cell counts, a decrease in the Th1/Th2 ratio, an increase in regulatory B cells, and a reduction in overall proinflammatory cytokine levels after BS (23). Despite lack of existing literature in this regard, NLR has been reported to decrease after laparoscopic adjustable band (24) and laparoscopic sleeve gastrectomy (LSG) (25). NLR significantly decreases within 3 months after LSG, along with a significant decrease in the levels of neutrophils and lymphocytes (25). However, there is little evidence to identify the changes in proportions of neutrophile and lymphocyte.

Our study aims to identify the longitudinal changes in the NLR of patients 6 months after sleeve gastrectomy by quantifying the neutrophil-lymphocyte ratio before and after the surgery. Furthermore, we intend to determine the relationship between NLR and weight loss as well as biochemical factors in patients with morbid obesity who underwent LSG.

## Materials and methods

This is a retrospective study in which clinical data of patients who underwent LSG between January 2019 and April 2021 were retrieved from electronic medical records. This study was approved by the Institutional Review Board of our institution **(EMRP22110N)** and was conducted following the Declaration of Helsinki. The institutional ethics committee waived the need for patients' written informed consent due to the retrospective nature of this study.

### Inclusion and exclusion criteria

We included patients who were admitted to our hospital and met the preoperative eligibility criteria for bariatric surgery. All included patients had data of complete blood cell count, white blood cell differential count, HbA1c, and HOMA-IR score one month before surgery, as well as three and six months post-operation. Patients less than 18 years with BMI less than 32.5 were excluded. Patients who presented with coexisting systemic inflammation, such as an ongoing infection, an unhealed venous ulcer, residual percutaneous drainage for infection diseases like cholecystitis, or pregnancy during the follow-up periods were excluded. Patients who had missing critical clinical data at postoperative 3- and 6-month follow-up, or had a postoperative complication within one month after operation which extended beyond the grade IIIa Clavien-Dindo classification [12], and patients who had revision surgery were excluded from the study.

### Procedure of LSG

The surgical procedure was the same for all patients at our institution. We inserted a 36-French oral gastral (OG) tube orally to serve as a guide during gastrectomy. Sleeve gastrectomy was performed from the antrum, 5 cm away from the pylorus to the angle of His along the OG tube. A staple line reinforcement was performed with a continuous oversewing of 3-0 polypropylene sutures before extracting the bougie. All patients were hospitalized for 24–48 h for postoperative observation. A cruroplasty with 2-0 ethibond thread and intraoperative endoscopic examination was routinely performed.

### Variables

In this study, excessive body weight is defined as total body weight-ideal body weight. In our institute, a BMI equal to 22 was defined as the ideal body weight. EBWL% was defined as the changes in excessive body weight. Diabetes was defined as having a preoperative hemoglobin A1c level >6.5% or the use of anti-diabetic medication or insulin injections. We defined hypertension as having a preoperative blood pressure level >140/90 mmHg or the use of antihypertensive medication. Dyslipidemia was defined as dyslipidemia detected by preoperative biochemical evaluation (One of the following criteria: Total cholesterol >200 mg/dl, Triglyceride ≥200 mg/dl, or LDL ≥ 140 mg/dl) or the use of medications for dyslipidemia. NLR was calculated by dividing the percentage of neutrophils by the percentage of lymphocytes from the white blood cell differential count. Insulin resistance for the patients was calculated using the following formula:


$$\left(\mathrm{HOMA}\right)-\mathrm{IR}=\frac{\;\mathrm{fasting}\;\mathrm{blood}\;\mathrm{glucose}\times \;\mathrm{fasting}\;\mathrm{insulin}}{405}$$

### Definition of successful weight loss at POM3

Previously, we had identified that excessive body weight loss (EBWL) > 19.5% in Postoperative 1- month (POM1) and >37.7% in postoperative 3-month (POM3) can successfully predict adequate weight loss, which was defined as EBWL > 50% at postoperative 6-month (POM6) (26). Moreover, these criteria have proven to be effective in predicting successful weight loss after LSG, when combined with rigorous lifestyle modifications such as a healthy balanced diet with guidance from a nutritionist, regular health check-ups, and software assistance (27). Thus, we use the EBWL > 37.7% as a definition of adequate weight loss at 3-month after LSG. Although it is not widely accepted, we believe that EBWL > 37.7% in POM3 is effective for predicting effect and help surgeons to do earlier additional intervention to increase effect after LSG.

### Measurement of clinical and biochemical data

Preoperatively(T0), we collected the patient's information including age, sex, past medical history, BMI, and comorbidities from the electronic medical records. We collected data on the body weight, biochemistry, and complete differential white blood cell count during postoperative outpatient follow-up at 3-month and 6-month to compare changes before and after the operative procedure. All biochemical measurements were performed in our hospital-accredited laboratory.

### Statistical analysis

All statistical analysis was performed by SPSS Version.22.0 (SPSS Inc., Chicago, IL) and MedCalc ver.19.1.5 (MedCalc Software Ltd., Ostend, Belgium). Continuous variables, including age, BMI, body weight loss, neutrophil and lymphocyte count, and the NLR were analyzed using the Kolmogorov-Smirnov test. Pre-and postoperative data were compared using the Wilcoxon rank-sum test (non-normal distribution) or Student's *t*-test (normal distribution). Results were expressed as mean ± standard deviation (normal distribution) or median value with interquartile range (IQR) (non-normal distribution). Differences among basic clinical characteristics, including gender and comorbidities, are listed in the contingency table, and compared using chi-square or Fisher's exact tests. The correlation between NLR and patient characteristics was examined using Pearson's correlation. Receiver operating characteristic (ROC) analysis was performed, and the cutoff point of NLR was calculated using the Youden index. A *p*-value <0.05 was considered significant.

## Results

Clinical data of 176 consecutive adult patients who fulfilled the inclusion criteria were collected between 2019 and 2021. We excluded 76 patients based on the exclusion criteria, including 49 patients with missing clinical data, one patient with postoperative staple line leakage who received rescue surgery, three patients who received revisional surgery, two patients with coexisting chronic wounds, one patient with percutaneous drainage for cholecystitis, one pregnant patient during the follow-ups, two patients who were less than 18-years-old and 17 patients who had a BMI < 32.5. Finally, a total of 100 patients were included in our study. The patient group consisted of 43 males and 53 females, the preoperative actual weight was 117.3 ± 24.1 kg in mean, BMI was 40.1 ± 7.9 in median, and the patient received 20.8 ± 8.0 months in mean of follow-up until the submission of the manuscript. Other relevant clinical parameters are shown in Table 1.

**Table 1:** Demographic data of included patients.

Total	*N* = 100
Age (yrs)	Mean (SD)	39.16 (11.23)
Sex (M/F)	(M/F)	44/56
Basic clinical characteristics.		
Weight (Kilograms)	Mean (SD)	117.33 (24.10)
Body mass index (BMI)	Median (IQR)	40.13 (7.87)
Follow up interval (months)	Mean (SD)	20.83 (8.04)
Hypertension	*n* (%)	63 (63%)
Diabetes Mellitus	*n* (%)	43 (43%)
Dyslipidemia	*n* (%)	36 (36%)

IQR, interquartile range; SD, Standard Deviation.

### Longitudinal changes in body weight and biochemical parameters

Table 2 demonstrates the longitudinal changes in body weight, biochemical parameters, and complete blood cell counts from preoperative(T0) to postoperative follow-up (POM3 and POM6). The median total body weight loss is 18.82% at POM3 and 26.94% at POM6. Compared to T0, body weight, insulin resistance, and levels of HbA1c, fasting insulin, insulin resistance, fasting C-peptide, triglyceride, hemoglobin, hematocrit, and platelets were significantly decreased at POM3 and POM6. However, body weight and triglyceride levels were significantly lower at POM6 than at POM3. In contrast, the C-peptide and high-density cholesterol levels were significantly higher at POM6 than at POM3. Other biochemical parameters demonstrated no differences between POM3 and POM6.

**Table 2:** Comparison of preoperative (T0) and postoperative (POM3 and POM6) changes in clinical parameters.

	Preoperative	POM3		POM6		
			*p* value (0 vs. 3M)		*p* value (3M vs. 6M)	*p* value (0 vs. 6M)
Bodyweight and Biochemical data						
Body weight (Kg) Mean (SD)	117.3 (24.10)	94.52 (18.98)	**<0**.001*	85.29 (16.35)	**<0**.001*	**<0**.001*
Excessive Body Wight Loss% Mean (SD)		42.96% (10.75%)	59.84% (12.91%)	** **	** **	** **
Total Body Weight Loss (%) median (IQR)		18.82% (5%)	26.94% (6%)	** **	** **	** **
HbA1c Median (IQR)	6.7 (6.1)	5.6 (5.5)	**<0**.001*	5.5 (0.5)	0.354	**<0**.001*
Insulin Median (IQR)	17.94 (13.6)	7.19 (6.99)	**<0**.001*	6.32 (5.33)	0.198	**<0**.001*
HOMA-IR Median (IQR)	4.55 (4.18)	1.68 (1.91)	**<0**.001*	1.35 (1.19)	0.102	**<0**.001*
C-peptide Mean (SD)	3.98 (1.51)	2.43 (0.84)	**<0**.001*	2.93 (1.63)	**0**.029*	**<0**.001*
TCH Mean (SD)	199.8 (36.2)	193.3 (36.7)	0.213	189.4 (35.8)	0.443	**0**.043*
TG Median (IQR)	159.5 (92)	96.0 (45.3)	**<0**.001*	78.0 (38.8)	**<0**.001*	**<0**.001*
HDL Median (IQR)	44.0 (15)	42.0 (11.8)	0.219	50.0 (14.8)	**<0**.001*	**<0**.001*
LDL Median (IQR)	126.5 (55.8)	120.0 (38.0)	0.852	117.0 (48.5)	0.199	0.189
HB Mean (SD)	14.1 (1.7)	13.4 (1.5)	**0**.003*	13.5 (1.5)	0.604	**0**.013*
HCT Mean (SD)	43.0 (5.3)	41.0 (4.0)	**0**.003*	41.4 (4.1)	0.536	**0**.015*
PLT*10^3^ Mean (SD)	296.9 (75.2)	270.1 (71.3)	**0**.010*	268.5 (64.0)	0.868	**0**.004*
ALB Median (IQR)	4.3 (0.3)	4.2 (0.3)	**0**.026*	4.2 (0.3)	0.501	**0**.**134**
White blood cell count						
Neutrophil to Lymphocyte ratio (NLR) Median (IQR)	2.21 (1.09)	1.78 (1.07)	**0**.005*	1.77 (0.88)	0.485	**<0**.001*
Definite White Blood Cell (/µl) Median (IQR)	8530 (3002.5)	6,830 (2357.5)	**<0**.001*	6,640 (2435)	0.784	**<0**.001*
Definite Neutrophil (/µl) Median (IQR)	5,369 (2190)	4,050 (1719)	**<0**.001*	4,045 (1937)	0.987	**<0**.001*
Definite Lymphocyte (/µl) Median (IQR)	2,440 (842)	2,100 (719)	**<0**.001*	2,185 (906)	0.294	**0**.003*

IQR, interquartile range; SD, Standard Deviation; TCH, Total cholesterol; TG, Triglyceride; HDL, high density cholesterol; LDL, low-density cholesterol; Hb, Hemoglobin; HCT, Hematocrit; PLT, Platelet; ALB, Albumin. *p < 0.05.

### Longitudinal changes in NLR, neutrophil, and lymphocyte count.

**Figure 1:**
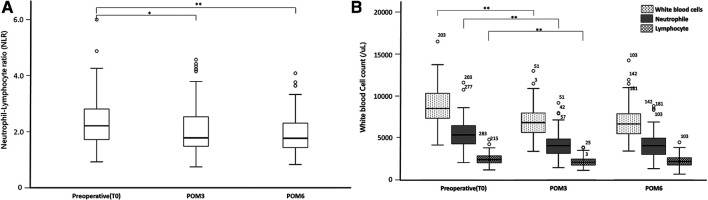
Changes in key serum leukocytes composition: (**A**) neutrophil to lymphocyte ratio (**B**) white blood cells, neutrophil counts and, lymphocyte counts. Significant differences were marked with the following thresholds: *p < 0.01, **p < 0.001.

The NLR decreased at POM3 (NLR_T0 vs. NLR_POM3: 2.21 vs. 1.78, *p* = 0.005) and POM6, compared to T0 (NLR_T0 vs. NLR_POM6: 2.21 vs. 1.77, *p* < 0.001) (Figure 1A). Similar to other biochemical data, the NLR did not change between POM3 and POM6 (NLR_POM3 vs. NLR_POM6: 1.78 vs. 1.77, *p* = 0.485) (Table 2).

Compared to T0, neutrophil and lymphocyte counts were both significantly lower at POM3 (Neutrophil_T0 vs. Neutrophil_POM3: 5369/µl vs. 4050/µl, *p* < 0.001, Lymphocyte_T0 vs. Lymphocyte _POM3: 2440/µl vs. 2100/µl, *p* < 0.001) and POM6 (Neutrophil_T0 vs. Neutrophil_POM6: 5369/µl vs. 4045/µl, *p* < 0.001, Lymphocyte_T0 vs. Lymphocyte _POM3: 2440/µl vs. 2185/µl, *p* = 0.003) (Figure 1B). However, there was no significant difference in neutrophil and lymphocyte counts between POM3 and POM6 (Neutrophil_POM3 vs. Neutrophil_POM6: 4050/µl vs. 4045/µl, *p* = 0.987, Lymphocyte_POM3 vs. Lymphocyte _POM6: 2100/µl vs. 2185/µl, *p* = 0.294).

Table 3 demonstrates the difference in neutrophil and lymphocyte counts between T0 and POM3, and between POM3 and POM6. At POM3, the declined amount (Neutrophil vs. Lymphocyte 1445.5/µl vs. 323.5/µl, *p* < 0.001) and the declined proportion (Neutrophil vs. Lymphocyte 25.11% vs. 13.07%, *p* < 0.001) of neutrophils was significantly higher than the lymphocytes. There were no significant differences between neutrophils and lymphocytes at POM6.

**Table 3:** Differences in preoperative (T0) and postoperative (POM3 and POM6) neutrophil and lymphocyte counts.

	PreOP-POM3	*p*-value	POM3-POM6	*p*-value
Difference in Neutrophil (/µl) Median (IQR)	1445.5 (1599)	**<0** **.** **001** *	31.87 (93)	0.827
Difference in Lymphocyte (/µl) Median (IQR)	323.5 (664)		−87.6 (461)	
Difference in Neutrophil% Median (IQR)	25.11% (29%)	**<0** **.** **001** *	−3.15% (38%)	0.866
Difference in Lymphocyte% Median (IQR)	13.07% (24%)		−4.74% (29%)	

IQR, interquartile range, *p < 0.05.

### Correlation of NLR with body weight change and biochemical profile

Table 4 demonstrates the correlation between NLR and clinical characteristics including body weight and biochemical profile. The NLR significantly positive correlated to the insulin level at T0 and POM3, as well as insulin resistance at POM3. The preoperative C-peptide, which indicates the functioning of the pancreas, showed a positive correlation with NLR at POM3. Also, higher preoperative HbA1c, which indicates poor pancreatic function and diabetes control, showed a negative correlation with the change in NLR between T0 and POM3. The NLR at T0 has a positive correlation to pre-and postoperative body weight. Moreover, NLR at T0 has a negative correlation with EBWL at POM3 and POM6.

**Table 4:** Correlation between the NLR and clinical characteristics.

	NLR_T0	ΔNLR_03	NLR_POM3	ΔNLR_36	NLR_POM6	ΔNLR_06
BW_T0	**0** **.** **217** *	0.144	0.195	0.098	0.087	**0**.224*
BMI_T0	0.144	**0**.244*	0.063	−0.067	0.105	0.172
TBWL_POM3	−0.15	0.137	−0.052	0.060	**−0**.247*	0.184
TBWL_POM6	−0.143	0.124	−0.063	0.012	**−0**.218*	0.129
EBWL_POM3	**−0**.217*	−0.034	−0.124	0.044	**−0**.272**	0.006
EBWL_POM6	**−0**.250*	−0.075	−0.149	0.010	**−0**.279**	−0.062
BW_POM3	**0**.250*	0.118	**0**.208*	0.085	0.137	0.188
BW_POM6	**0**.261**	0.105	**0**.224*	0.099	0.154	0.188
BMI_POM3	0.175	**0**.203*	0.072	−0.084	0.161	0.119
BMI_POM6	0.191	**0**.202*	0.086	−0.078	0.184	0.123
TCH_T0	−0.056	0.102	−0.071	−0.118	0.006	−0.008
TCH_POM3	−0.023	−0.013	0.035	0.012	0.052	−0.002
TCH_POM6	−0.07	0.046	−0.047	−0.098	0.110	−0.043
TG_T0	0.176	0.016	0.121	0.082	−0.032	0.089
TG_POM3	0.151	−0.006	0.178	0.012	0.178	0.005
TG_POM6	0.068	−0.040	0.16	0.085	0.160	0.038
HDL_T0	−0.008	0.010	0.09	−0.059	0.090	−0.042
HDL_POM3	−0.084	−0.194	−0.081	0.083	−0.081	−0.111
HDL_POM6	−0.189	−0.182	−0.116	0.009	−0.116	−0.165
LDL_T0	−0.154	0.097	0.042	−0.175	0.042	−0.063
LDL_POM3	−0.034	0.071	0.029	−0.053	0.076	0.020
LDL_POM6	−0.038	0.109	−0.017	−0.130	0.127	−0.012
Insulin_T0	**0**.253*	1.171	0.239	0.023	0.196	0.198
Insulin_POM3	0.055	**−0**.314**	**0**.315**	**0**.279*	0.152	**−0**.**046**
Insulin_POM6	0.006	−0.064	0.143	−0.044	0.176	−0.108
HIR_T0	0.237	0.078	0.222	0.107	0.059	0.184
HIR_POM3	0.056	**−0**.297*	**0**.311**	**0**.254*	0.130	−0.053
HIR_POM6	0.018	−0.038	0.116	−0.044	0.155	−0.082
A1C_T0	0.013	**−0**.210*	0.157	0.066	0.007	−0.142
A1C_POM3	−0.017	−0.106	0.697	−0.058	−0.027	−0.152
A1C_POM6	−0.002	−0.056	−0.018	−0.054	−0.059	−0.101
Cpep_T0	0.194	0.147	**0**.218*	0.111	0.056	**0**.239*
Cpep_POM3	0.137	**0**.470*	−0.216	0.143	−0.228	**0**.487**
Cpep_POM6	0.115	−0.009	0.044	0.034	0.111	0.021
ALB_T0	0.125	0.031	0.376	**0**.216*	−0.081	**0**.222*
ALB_POM3	0.096	−0.184	0.157	**0**.256*	0.002	0.053
ALB_POM6	0.148	−0.082	0.087	0.155	0.020	0.060

ΔNLR, The change of NLR; BW, Body weight; BMI, Body mass index; TBWL, Total body weight loss; EBWL, Excessive body weight loss; TCH, Total cholesterol; TG, Triglyceride; HDL, high density cholesterol; LDL, low density cholesterol; ALB, Albumin. *p < 0.05, **p < 0.01.

### Prediction of adequate weight loss (EBWL > 37.7%) at POM3 by preoperative NLR

Previously, we had identified that excessive EBWL at POM1 > 19.5% and EBWL at POM3 > 37.7% can successfully predict adequate weight loss, which was defined as EBWL > 50% at POM6 [26]. Thus, we use EBWL at POM3 > 37.7% as a definition of adequate weight loss three months after LSG.

To evaluate the prognostic accuracy, receiver operating characteristic curve (AUC) analysis was used to evaluate the predictive capability of NLR in EBWL at POM3 > 37.7%. After calculation, NLR at T0 showed significant predictive accuracy for successful weight loss [EBWL at POM3 > 37.7%; AUC 0.635 (95% CI: 0.532–0.729), *p* = 0.032] with a sensitivity of 67.6%, and specificity of 62.5%. According to the Youden index, the cutoff value of preoperative NLR < 2.36 was able to predict successful weight loss at 3-month.

## Discussion

In this study, we demonstrated the longitudinal changes in NLR, neutrophil, and lymphocyte counts before and at three and six months after LSG. In summary, NLR significantly decreased between T0 and POM3 whereas no significant change was observed between POM3 and POM6. The neutrophil and lymphocyte count significantly decreased from T0 to POM3 but did not change between POM3 and POM6. The counts and proportion of neutrophils decreased more significantly than that of the lymphocytes. The NLR at T0 negatively correlated with EBWL at POM3. After ROC analysis, NLR at T0 showed a significant predicting accuracy in EBWL at POM3 > 37.7%. (AUC 0.635, *p* = 0.032) at a cutoff value of 2.36.

BS is currently one of the most effective therapeutic modalities for morbid obesity and produces significant weight loss and improvement in glucose metabolism. Compared with lifestyle modifications, and restrictive dietary interventions, bariatric surgery offers a significant weight reduction, reduces obesity-associated comorbidities, increases the probability of pregnancy (HR = 2.886, *p* < 0.001)as well as successful vaginal birth (HR 6.426, *p* < 0.001) (28), and lowers the risk of respiratory tract infection (HR = 0.432, *p* < 0.001) (29), malignancy(30) and weight regain. Laparoscopic sleeve gastrectomy (LSG) is deemed to be the most popular bariatric procedure in the world in recent years. Obesity-induced inflammation is associated with a higher incidence of malignancies (31). Post bariatric surgery, systemic inflammation decreases as indicated by a decrease in C-reactive protein levels (32–37). Meanwhile, levels of high-sensitivity C-reactive protein and complement components 3 and 4 in serum significantly decrease in obese patients who underwent LSG (38). The NLR, which is indicated as a systematic inflammation maker, also decreases after LSG. Our findings are consistent with that of previous research which demonstrated a decline in NLR in patients with obesity after laparoscopic adjustable band (24) and laparoscopic sleeve gastrectomy (LSG) (25).

Concerning the relationship between obesity, baseline NLR, and systemic inflammation, the NLR significantly decreased within 3-months after LSG along with a significant decrease in neutrophils and lymphocytes like previously published data (25). The decline of NLR may be attributed to a simultaneous decline in subclinical systemic inflammation after bariatric surgery. However, we performed further analysis and identified that neutrophils had a more profound change between T0 and POM3 compared to lymphocytes. The change in lymphocyte function after bariatric surgery is well identified. B-cell activating factors, including APRIL, BAFF, and soluble CD40L, as well as systematic inflammatory factors, including high sensitivity CRP, IL-1β, IL-12, IL-18, and IFN-*γ*, decrease after bariatric surgery. These changes may lead to a decrease in B-cell activity, IgG levels, and systemic inflammation (39). Meanwhile, a reduction in CD4+ and CD8+ T cell counts and a decrease in the Th1/Th2 ratio were also identified after bariatric surgery (23). Moreover, following the regulation of blood sugar levels and control of diabetes, immune function improves and this is demonstrated by a significant increase in lymphocytes (CD3+), killer T cells (CD3 + CD8+) and B cell subsets (CD19 + CD45+) (40). Additionally, an improvement in immunity following LSG may offer protection against cancer for patients with obesity. For example, IL-1β regulates the expression of adipocyte-mediated vascular endothelial growth factor A and angiogenesis, which may lead to the progression of breast cancer (41). Previously published data have shown that higher NLR is associated with an increased risk of breast cancer among the Asian population (6). Our recent study also found a decrease in the incidence of malignancy in patients who had bariatric surgery (30). From previously published studies, bariatric surgery can be employed as a cancer prevention method for patients with morbid obesity although the definite mechanism in the role of obesity-induced chronic inflammation in oncogenesis is still vague.

It is known that neutrophils are the first immune cells to respond to inflammation and can promote a chronic inflammatory state by recruitment of macrophages and interaction with antigen-presenting cells (42–44). High-fat diets induce neutrophil infiltration into adipose tissue as demonstrated by Elgazar-Carmon V et al., who found that neutrophils transiently infiltrated the parenchyma of intra-abdominal adipose tissue three days after initiation of a high-fat diet in C57BL/6J mice. Compared with day 0, the mean periepdidymal fat myeloperoxidase expression, which represents neutrophils, was significantly increased by 3.5-fold (*P* < 0.01) on day 3 and 2.9-fold (*P* < 0.03) on day 7 and was indistinguishable up to day 28 (45). After infiltrating the adipocytes, the secreted elastase from neutrophils may further exacerbate the inflammatory process, leading to enhance macrophage recruitment resulting in chronic inflammation and insulin resistance (46). The inflamed adipocyte, along with the nearby macrophages, may generate IL-8 (47, 48) which acts as a local chemoattractant for neutrophils and T cells. On the other hand, the production of leukotriene B4 in white adipose tissue also contributes to neutrophil accumulation. Moreover, the crosstalk between neutrophils and adipocytes may generate IL-1β expression *via* the NF-κB pathway to promote adipose tissue inflammation (49). In summary, obesity induces neutrophil activation and promotes chronic inflammation *via* this mechanism.

Neutrophils influence the oncogenesis and prognosis of cancer. It is been demonstrated that cancer cells can cause endoplasmic reticulum stress in the peritumor neutrophils transforming them into Lectin-type oxidized LDL receptor 1 (LOX1)-expressing immunosuppressive neutrophils (50). The LOX-1+ neutrophils may suppress T-cell function and induce tumor progression (51). However, there is a paucity of data to evaluate the function of neutrophils in patients with obesity as well as its influence on oncogenesis. Roberts HM et al. reported that the peripheral blood neutrophils from patients with obesity have higher ROS generation, decreased NET formation and chemotaxis, and an enhanced cytokine secretion all of which lead to local and systemic inflammation. Weight loss after gastric band increases NET formation and chemotaxis, decreases ROS production and cytokine release (52). Further studies on the relationship between changes in neutrophil function following bariatric surgery and oncogenesis are needed.

In our institution, most of the patients had successful weight loss following bariatric surgery. However, a subgroup of the patients had a suboptimal weight loss after bariatric surgery and were more prone to weight regain. Therefore, a biomarker that could predict the outcome of bariatric surgery at an earlier timepoint is of utmost clinical relevance. Previously, we had identified a scoring system, 6M50LSG, which proved that excessive body weight loss in Postoperative 1- month (EBWL_POM1) > 19.5% and excessive body weight loss in Postoperative 3-month (EBWL_POM3) > 37.7% can successfully predict adequate weight loss, which was defined as EBWL > 50% at postoperative 6-month (EBWL_POM6) (26). Moreover, we have proven that these criteria combined with medical nutrition therapy (MNT) can significantly elevate the success rate of laparoscopic sleeve gastrectomy (27). However, among patients who were predicted as poor responders and received upgraded medical nutrition therapy, 52% had inadequate weight loss. Additional factors that contribute to suboptimal responses of patients to bariatric surgery should be identified. In this study, we demonstrated that NLR has a negative correlation and possesses a prognostic power in predicting excessive body weight loss at an earlier postoperative period based on baseline NLR levels. Moreover, we identified that preoperative NLR can predict the successful outcome at 3-month (EBWL_POM3 > 37.7%). Our findings provide reliable information for bariatric surgeons to implement interventions immediately after surgery to optimize weight loss and metabolic outcomes in patients who are expected to be poor responders to bariatric surgery. However, further study should be designed to evaluate the value of prediction in successful outcome.

The study also has its limitation. Firstly, it is a retrospective study, and most of the data were acquired from electronic medical records in our hospital. Despite our best effort, bias and missing relevant data were inevitable. Also, our cohort was limited to the Asian population, with pre-defined BMI, age, and sex composition, which may be incapable to represent the entire cohort of morbid obesity patients who are indicated for bariatric surgery. Moreover, our cohort has a relatively short duration of follow-up, warranting the need for more studies with long-term follow-ups to assess the correlation between NLR and long-term bariatric outcomes. Lastly, due to the lack of analysis of other important inflammatory markers such as CRP (53), IL-1, IFN-*γ*, IL-6 and TNF-α (54, 55), a thorough understanding of the interaction between changes in body weight and the immune response is limited.

## Conclusion

In conclusion, our study demonstrated that NLR significantly decreased between T0 and POM3 with no significant change between POM3 and POM6. The neutrophil and lymphocyte count significantly decreased from T0 to POM3 but were similar between POM3 and POM6. Furthermore, neutrophils had a more profound significant decline compared to lymphocytes during the follow-up at 3-month. The NLR at T0 has a negative correlation to EBWL at POM3. As an early prognostic marker, pre-operative baseline NLR < 2.36 can predict successful weight loss (EBWL > 37.7%), after three months of bariatric surgery. Further analysis of the relationship between obesity, the outcome of bariatric surgery, neutrophil function, and oncogenesis should be made to validate the findings of this study.

## Data Availability

The raw data supporting the conclusions of this article will be made available by the authors, without undue reservation.
